# The Factor Structure of Intimate Partner Violence Risk

**DOI:** 10.1177/00938548251357789

**Published:** 2025-09-01

**Authors:** Anna T. Pham, Kevin L. Nunes, N. Zoe Hilton, Liam Ennis, Sandy Jung

**Affiliations:** Carleton University; Department of National Defence; Carleton University; University of Toronto; Waypoint Research Institute; University of Alberta; MacEwan University

**Keywords:** intimate partner violence, domestic violence, exploratory factor analysis, factor structure, risk assessment

## Abstract

To improve our understanding of the latent constructs of intimate partner violence (IPV) risk, we explored the underlying factor structure of combined items from three IPV risk assessment tools and examined whether the factors predict recidivism outcomes. Data were examined for 251 adult men who were charged with violence against their past or current female intimate partners and whose files were referred for a comprehensive threat assessment from 2010 to 2016 in Canada. Results suggested six underlying risk factors, two of which significantly predicted IPV, any violent, and any recidivism outcomes in a 4-year average follow-up with 227 men. However, only one factor (*Antisocial Patterns and Psychosocial Adjustment*) independently predicted IPV and any violent recidivism over time above and beyond other factors. Our findings indicate room to further improve current IPV risk assessment measures and support the call for informative causal theories of IPV recidivism.

Intimate partner violence (IPV)—generally defined as the actual, attempted, or threatened physical harm of a current or former intimate partner—represents the largest single category of calls to police in North America (e.g., Burczycka et al., 2018; Klein, 2009). However, knowledge gaps remain about how best to intervene in IPV cases, particularly given the finite resources available to the criminal legal system. Considerable research demonstrates that the effects of criminal legal and therapeutic interventions are maximized when their intensity is determined by the level of risk (e.g., Risk-Need-Responsivity [RNR] principles; Bonta & Andrews, 2024). Effective risk assessment helps identify persons who are in the highest risk category and thus is considered the cornerstone of offender management (Bonta & Andrews, 2024). Thus, optimizing IPV risk assessment tools is imperative to enhancing IPV risk assessment and risk management.

Theoretical understandings of violence risk are important to the development of risk assessment instruments, yet the available theories on IPV recidivism have been noted for their failure to produce predictive models (Bell & Naugle, 2008). Although the literature contains a plethora of theories on factors contributing to the onset of IPV, theories on risk factors predictive of repeated IPV (or IPV recidivism) remain scarce. For example, Hilton (2021) notes that risk factors that explain why people commit IPV in the first place are not necessarily useful for distinguishing who—among men who have encountered the criminal legal system for IPV—will recidivate. Instead, researchers must rely on risk factors from empirical research, literature reviews, or clinical observations to develop risk assessment tools for IPV recidivism. While this approach is helpful to identify the strongest predictors of IPV recidivism, it does little to further our understanding of the relationship between the latent factors that underlie these risk items and recidivism. Furthermore, it is unclear whether commonly used IPV risk tools contain overlapping risk constructs, as well as the most relevant risk information to reliably predict recidivism. Until well-established theories are available to inform IPV risk assessment, researchers must use a bottom-up approach to understand the underlying factor structure of IPV risk, which would then inform new or refine current theories of IPV recidivism. Therefore, the present study aims to explore the factor structure of commonly used IPV risk assessment measures and examine whether the underlying risk factors that comprise them are strongly related to, and predict, IPV recidivism.

## IPV Risk Assessment Measures and Their Factor Structure

Several violence risk assessment tools have been developed to assess the risk of repeat IPV. One meta-analysis identified 39 different measures that were either developed for or applied to IPV risk assessment (van der Put et al., 2019), with the Ontario Domestic Assault Risk Assessment (ODARA; Hilton et al., 2004) and Spousal Assault Risk Assessment guide (SARA; Kropp & Hart, 2000) being among the most used tools. The ODARA is a 13-item risk assessment tool (see Table 1 for full list of items) designed for use by frontline police officers and other workers at the scene of an IPV call (Rice et al., 2010) to evaluate the risk of man-to-woman IPV recidivism among men who have been identified as having committed at least one act of IPV (Hilton, 2021; Hilton et al., 2004; Hilton, Harris, & Rice, 2010). The tool has good predictive validity for IPV recidivism with AUCs ranging from .65 to .74 in individual studies and meta-analyses (Hilton et al., 2008; Hilton & Harris, 2009; Messing & Thaller, 2013; van der Put et al., 2019).

**Table 1:** List of ODARA, SARA, and B-SAFER Items

ODARA	SARA (section)	B-SAFER (section)
1. Pre-index domestic assault in a police or criminal record 2. Pre-index nondomestic assault in a police or criminal record 3. Pre-index custodial sentence of 30 days or more 4. Failure on pre-index conditional release 5. Threat to physically harm or kill at the index assault 6. Confinement of the partner at the index assault 7. Victim concern about future assaults 8. More than one child altogether 9. Victim’s biological children from a previous partner 10. Pre-index violence against a nondomestic victim 11. Two or more indicators of substance abuse 12. Assault on index victim when pregnant 13. Barriers to victim support	1. Past assault of family members (CH) 2. Past assault of strangers or acquaintances (CH) 3. Past violation of conditional release or community supervision (CH) 4. Recent relationship problems (PA) 5. Recent employment problems (PA) 6. Victim of and/or witness of family violence as a child or adolescent (PA) 7. Recent substance abuse/dependence (PA) 8. Recent suicidal or homicidal ideation/intent (PA) 9. Recent psychotic and/or manic symptoms (PA) 10. Personality disorder with anger, impulsivity, or behavioral instability (PA) 11. Past physical assault (SAH) 12. Past sexual assault/sexual jealousy (SAH) 13. Past use of weapons and/or credible threats of death (SAH) 14. Recent escalation in frequency or severity of assault (SAH) 15. Past violation of “no contact” orders (SAH) 16. Extreme minimization or denial of spousal assault history (SAH) 17. Attitudes that support or condone spousal assault (SAH) 18. Severe and/or sexual assault (CO) 19. Use of weapons and/or credible threats of death (CO) 20. Violation of “no contact” order (CO)	1. Violent acts (IPV) 2. Violent threats or thoughts (IPV) 3. Escalation (IPV) 4. Violation of court orders (IPV) 5. Violent attitudes (IPV) 6. General criminality (PA) 7. Intimate relationship problems (PA) 8. Employment problems (PA) 9. Substance use problems (PA) 10. Mental health problems (PA) 11. Inconsistent attitudes or behavior (VV) 12. Extreme fear of perpetrator (VV) 13. Inadequate support or resources(VV) 14. Unsafe living situation (VV) 15. Health problems (VV)

*Note.* ODARA = Ontario Domestic Assault Risk Assessment; SARA = Spousal Assault Risk Assessment; B-SAFER = Brief Spousal Assault Form for the Evaluation of Risk; CH = Criminal History section; PA = Psychosocial Adjustment section; SAH = Spousal Assault History section; CO = Current Offence section; IPV = Intimate Partner Violence section; VV = Victim Vulnerability section.

The ODARA is an actuarial tool developed through empirical research and interpreted using statistical data with minimal theoretical influence on the potential predictors studied. Items in actuarial risk assessment tools are predetermined and selected because they are easily accessible and have been empirically demonstrated to increase risk of recidivism through follow-up studies. These tools also have explicit rules for scoring individual items, and there is typically an algorithm for obtaining a total score related to numeric risk of recidivism in actuarial tables. Correspondingly, the ODARA items have good predictive value but limited explanatory value.

By contrast, the SARA and the abbreviated Brief Spousal Assault Form for the Evaluation of Risk (B-SAFER) are structured professional judgment (SPJ) tools comprising risk factors rationally derived from literature reviews and clinical considerations (Kropp & Hart, 2000; Kropp et al., 1999). These tools typically contain a structured list of predetermined risk factors that are empirically or theoretically related to recidivism, but the method of combining items into a total score is not specified, and the overall risk evaluation is left to the professional judgment of the evaluator (Boer et al., 1997). The SARA was developed as an inventory of risk factors for IPV cases (Kropp et al., 1995, 1999) and contains 20 items divided into five content areas (referred to as *sections*; see Table 1 for full list of items). The SARA has consistently demonstrated good predictive validity with file information (e.g., Belfrage et al., 2012; Hilton et al., 2004; Jung & Buro, 2017; Olver & Jung, 2017), and its total score predicts repeat IPV with an average AUC of .63 (Helmus & Bourgon, 2011; Messing & Thaller, 2013).

The B-SAFER is a 15-item (see Table 1 for full list of items) version of the SARA designed for use by police officers (Kropp & Hart, 2004) and was developed with the purpose of improving upon the limitations of the SARA. Empirical studies have found some support for its predictive validity. For example, the summed risk score predicted any recidivism in 40 men imprisoned for an offense against an intimate partner (AUC = .76; Loinaz, 2014). However, it did not significantly predict IPV recidivism in 158 men court-ordered to treatment for violence or stalking (AUC = .55; Gerbrandij et al., 2018). The SARA and B-SAFER are organized with a focus on their practical utility in the field rather than based on the authors’ conceptualization of an underlying factor for IPV risk.

Currently, risk of IPV recidivism is understood as involving distinct constructs (e.g., McDonagh et al., 2024), but the precise number and nature of these constructs are unclear. For example, the SARA and B-SAFER items are categorized into four and three sections, respectively (see Table 1), suggesting that both measures contain multiple underlying risk factors. While the factor structure of the SARA has been empirically examined by Hilton, Harris, and Rice (2010), the B-SAFER’s factor structure has yet to be empirically investigated in the literature.

Only two published studies have empirically examined the factor structure of IPV risk measures. Hilton, Harris, and Rice (2010) conducted an exploratory factor analysis (EFA) of combined items from the ODARA, Danger Assessment (Campbell, 1986), SARA, Domestic Violence Screening Instrument (Hisashima, 2008), and Psychopathy Checklist–Revised (Hare, 1991). They found four distinct underlying risk factors. Items in the first factor reflected attitudes toward IPV, plus socio-affective, emotional, and interpersonal aspects of psychopathy. Items in the second factor reflected antisocial patterns (e.g., general criminality, substance abuse, prior arrests). Items in the third factor reflected IPV-related technical violations. Items in the fourth factor reflected threats, use of weapons, and sexual assault items from all included measures. Pham and Jung (2022) factor analyzed ODARA items using a sample of 234 men charged for a violent offense against their past or current female intimate partners and found that the measure comprised three distinct factors related to antisocial patterns, victim vulnerabilities, and index offense.

Both Hilton, Harris, and Rice (2010) and Pham and Jung (2022) found that factors reflecting the antisocial patterns construct strongly predicted IPV recidivism, consistent with prior research demonstrating the utility of the antisocial patterns construct to predicting IPV recidivism (e.g., Fitzgerald & Graham, 2016; Hilton & Radatz, 2021; López-Ossorio et al., 2017). However, both studies found that many of the other underlying risk factors were unrelated to IPV recidivism, such as those reflecting characteristics of the index offense and victim circumstances. These findings are inconsistent with the literature (e.g., Connor-Smith et al., 2011; López-Ossorio et al., 2017), underscoring the need to not only explore the latent constructs that comprise commonly used risk assessment tools but also to examine whether they are relevant for the prediction of IPV recidivism.

## The Present Study

Given the popularity of the ODARA and SARA in IPV risk assessment (van der Put et al., 2019), as well as the development of the B-SAFER (and ODARA) for frontline use (Kropp & Hart, 2004), the aim of the present study was to shed light on the factor structure of IPV risk as measured by these three tools. To that end, first, we factor analyzed their combined items. Next, we examined whether the resulting factors significantly predicted general, any violent, and IPV recidivism, using a series of receiver operating characteristic (ROC) and Cox regression analyses. Given past findings of at least three latent factors that comprise IPV risk assessment measures (i.e., Hilton, Harris, & Rice, 2010; Pham & Jung, 2022), we hypothesized that the EFA would also reveal at least three factors. We also expected that few factors would significantly predict recidivism outcomes. Relatedly, we expected that factors reflecting antisocial patterns would significantly and independently predict recidivism outcomes better than other factors.

## Method

### Sample

Our sample consisted of 300 adult men charged or accused of violence against their past or current women intimate partners and whose files were referred between 2010 and 2016 to a comprehensive threat assessment service in Canada (Ennis et al., 2015). The men’s mean age was 35.3 (*SD* = 9.0, range = 18–65). Most were identified as White (*n* = 189, 63.0%), followed by Indigenous (*n* = 81, 27.0%), with the remainder identified as other ethnicities (*n* = 27, 9.0%; *n* = 3, 1.0% missing). The mean highest grade completed was 10.5 (*SD* = 2.2), with only 34.0% (*n =* 102) having completed high school. Almost half were unemployed at the time of their index offense (*n* = 134, 44.4%). Half were married or cohabiting with the victim at the time of the index (*n* = 154, 51.3%), 27.0% (*n* = 81) were formerly married or cohabiting, 13.0% (*n* = 39) were currently dating, and 7.3% (*n* = 22) were previously dating (*n* = 4, 1.3% missing). Most relationships had lasted at least 24 months (*n* = 185, 61.7%).

### Measures

#### Ontario Domestic Assault Risk Assessment

The Ontario Domestic Assault Risk Assessment’s (ODARA) 13 items (see Table 1) are scored dichotomously as “1” *Present* or “0” *Not Present* and summed to generate a total risk score, ranging from 0 to 13. The ODARA is intended to be scored from police and criminal justice records with or without a victim interview. Studies demonstrate that the ODARA has poor to acceptable internal consistency (α = .40–.65; Hilton et al., 2008, 2021) and can be scored reliably from archival data with intra-class correlations (ICCs) ranging from .84 to .95 (Hilton, Harris, Popham, et al., 2010; Hilton & Radatz, 2021). In addition, Hilton and colleagues (2008, 2021) found significant positive relationships between the ODARA total score with SARA version 2 (*r* = .60, *p* < .01) and version 3 (*r* = .48, *p* < .01), B-SAFER (*r* = .40, *p* < .01), DA (*r* = .43, *p* < .001), and DVSI (*r* = .52, *p* < .001).

#### Spousal Assault Risk Assessment

The Spousal Assault Risk Assessment’s (SARA) 20 items (see Table 1) are scored using a 3-point nominal scale, where “0” indicates *No/Absent*, “1” indicates *Possible/Partially Present*, and “2” indicates *Yes/Present*. Based on the scoring of the 20 items, the rater makes a summary risk judgment indicating that the individual is at low-, moderate-, or high-risk for committing future IPV. Although the formal use of the SARA involves an overall judgment of future IPV risk by the clinician that is guided by the presence or absence of items, most studies have quantified and summed the items into a total score, and Kropp and Hart (2000) found that the SARA had excellent interrater reliability (IRR) of summed scores (ICC = .84) and good internal consistency (α = .78).

#### Brief Spousal Assault Form for the Evaluation of Risk

The Brief Spousal Assault Form for the Evaluation of Risk’s (B-SAFER) 15 items (see Table 1) are also scored using the same 3-point scale as the SARA. As with the SARA, assessors score the B-SAFER items, which then inform a summary rating of risk. Note that the B-SAFER also includes *past* (i.e., over 4 weeks prior to assessment) and *recent* (i.e., during the 4 weeks prior to assessment) for each item, resulting in a total of 30 items. For the purposes of examining factor structures in the present study, however, B-SAFER Recent items were excluded because information during the 4 weeks prior to assessment was often unavailable, resulting in poor IRR (ICC = .22; Hilton et al., 2021). Like the SARA, the authors recommend the B-SAFER to be used as a guide, rather than as a mechanical measure. To date, there is little published research on the B-SAFER, but the available studies (e.g., Gerbrandij et al., 2018; Loinaz, 2014; Storey et al., 2014; Svalin & Levander, 2020) suggest that the measure has reasonable IRR.

#### Recidivism

We coded criminal record data from local, provincial, and federal sources retrieved from 2016 to 2020, specifically incidents following the date of release from custody. We used three recidivism outcomes: (a) Any new convictions and/or charges, excluding supervision or technical violations (Any Recidivism), (b) any new violent convictions and/or charges (e.g., assault) against anyone (e.g., strangers, acquaintances, partners, family members, etc.; Any Violent Recidivism), and (c) any new violent convictions and/or charges against a past or current intimate partner (including, but not limited to, the index partner, which is appropriate since the tools we used were not designed to assess risk against only one victim; Any IPV Recidivism). The average follow-up was 4 years (*SD* = 1.50) and ranged from 0.94 to 7.86 years.

### Procedure and Sources of Information

This research was approved by the research ethics boards at the authors’ four primary institutions. A formal notice of collaboration was obtained from the Integrated Threat and Risk Assessment Centre where the study took place. We identified 300 cases referred by police for threat assessment between 2010 and 2016 based on two criteria, such that cases must have: (1) full or provisional threat assessment reports to ensure that sufficient documents and information were available to reliably extract, and accurately code, data, and (2) an identifiable index offense that matched with the ODARA-defined index offense (Hilton et al., 2004). From 2015 to 2021, all cases were coded for the ODARA, SARA, and B-SAFER items, and recidivism outcomes (see Measures) by undergraduate and graduate research assistants who completed standard online training on all three measures (see Hilton & Ham, 2015, for the ODARA training description).

While coders were blind to information on reoffending and other relevant behaviors after the assessment date, they had access to the same case file information that threat assessors used for their evaluation. These files included a referral form (e.g., demographic information, historical occurrences of IPV), official criminal records, and police occurrence documents (e.g., investigator notes, documented evidence, arrest details). In addition, reports by the assigned certified threat assessor typically included a case history (e.g., childhood, employment, substance use history), list of criminal convictions/sentences, intimate relationship history including associated violence, case management recommendations, and risk assessment (e.g., ODARA, SARA, or both). Some files also included reports from mental health agencies and victim interview documentation. To identify any post-index violent and nonviolent offenses at follow-up, we retrieved criminal records up to 2020, and the corresponding police occurrence reports for any post-index violent charges to identify whether the victim was an intimate partner.

To ensure continued adherence to coding instructions and avoid coding drift or complacency, coders were tested for coding accuracy on at least one case previously coded by the authors and at least one subsequent test case (also previously coded by the authors). These procedures, including examination of IRR with 32 cases, have been described in a previous publication (Hilton et al., 2021). Interrater reliability (ICC) was above .80 for ODARA and B-SAFER items (past items only; see Measures) and between .70 and .79 for SARA items.

### Statistical Analyses

We explored the factor structure of the ODARA, SARA, and B-SAFER Past items using EFA in MPlus Version 8.5 (Muthén & Muthén, 2015). First, we analyzed a polychoric correlation matrix of the measured items to reveal any underlying processes in the scales (Floyd & Widaman, 1995) because polychoric correlations are ideal with linearly related, ordinal, and binary variables (Flora & Curran, 2004; Holgado-Tello et al., 2010). We then used the weighted least square estimator method to extract factors as it is robust against small sample sizes, any violations of normality (Flora & Curran, 2004) and performs well regardless of ceiling and floor effects in ordinal data with sample sizes as small as 200 (Brown, 2006). For factor retention, we examined the scree plot, Kaiser’s criterion, parallel analysis, and Velicer’s minimum average partial test. To maximize interpretability, we used the oblique rotation method, which computes the degree to which items load onto factors (Tabachnick & Fidell, 2019).

We used general rules regarding cutoff values as guidelines for factor structure fit: root mean square error of approximation (RMSEA; no greater than .06; Hu & Bentler, 1999), comparative fit index (CFI; .95 or greater; Brown, 2006), and standardized root mean square residual (SRMR; no greater than .08; Hu & Bentler, 1999; Schmitt, 2011). In addition to placing items with factor loadings of .30 to .40 or greater onto a factor (Cudeck & O’Dell, 1994; Schmitt & Sass, 2011), we also investigated whether the standardized factor loading significantly differed from zero to assess whether an item significantly loaded onto a factor (Cudeck & O’Dell, 1994; Schmitt, 2011; Schmitt & Sass, 2011). To determine the significance and protect against Type 1 error, a correction procedure for correlated factors was used to compute the appropriate α level (see Cudeck & O’Dell, 1994; Schmitt, 2011). The *z*-score associated with the determined α level was then used as the critical point for determining significance. To use the retained factors in subsequent analyses, we generated two composite variables that provide information on an individual’s placement or ranking on the factors (DiStefano et al., 2009): (1) Factor Scores (generated with MPlus), which were used in subsequent AUC and Bootstrap analyses, and (2) Factor Sums (generated by summing all item scores in a given factor), which were used in subsequent Cox regression analyses for interpretability purposes.

We generated AUC statistics to examine each factor’s predictive validity for predicting all recidivism outcomes via the ROC analyses in SPSS version 27. AUC is the most used and recommended effect size statistic for evaluating the prediction of recidivism and is typically preferred to other measures of predictive accuracy that are more affected by recidivism base rates (Helmus & Babchishin, 2017; Rice & Harris, 2005). AUC values are traditionally interpreted as the probability of a recidivist scoring higher than a non-recidivist on a given measure. Generally, AUCs above .50 indicate prediction exceeding chance and AUCs of above .55, .63, and .70 correspond to small, moderate, and large effects, respectively (Rice & Harris, 2005).

Next, we conducted pairwise comparisons of the AUCs to test for significant differences in the factors’ predictive validity, using a Bootstrap test (Carpenter & Bithell, 2000). ROC analyses were generated using the pROC statistical package in R (Robin et al., 2011) and all statistical analyses were based on the nonparametric bootstrapping method (Carpenter & Bithell, 2000), with 10000 replications, to test for significant differences in AUCs of two ROC curves. In each replication, the original measurements were resampled with replacement, where the same number of case and control observations as in the original sample were selected in each bootstrap replicate. Both new ROC curves corresponding to this new sample were built, and the resampled AUCs and their difference, *D*, were computed, which represents the bootstrap statistics for comparing AUCs. Given that only Factors 2 and 3 significantly predicted all recidivism outcomes, we compared these two factors against all other factors, as well as with each other, to avoid increasing the risk of Type I error unnecessarily. We used Bonferroni correction to account for conducting nine pairwise comparisons for each recidivism outcome, in which a difference in two AUCs would only be considered as statistically significant if *p* < .006 (.05/9 = .006).

In addition, we conducted Cox regression analyses in SPSS version 27 to test the incremental predictive validity of each EFA factor. We computed the length of time until recidivism from the date of release from custody to the first date of post-index arrest or charge for a new IPV offense and any new violent offense for survival analyses. Incremental prediction was not tested for any recidivism because the date of the first general offense was not coded.

## Results

### Factor Structure of IPV Risk Measures

We entered 48 items from the ODARA, SARA, and B-SAFER Past items in the EFA to assess their underlying factor structure. Eleven items violated the multicollinearity assumption and were subsequently removed from the EFA, resulting in a final inclusion of 37 items in the EFA. Note that our sample size is smaller than previously recommended (e.g., Comrey & Lee, 1992; Tabachnick & Fidell, 2019); however, we conducted the Kaiser–Meyer–Olkin (KMO; Kaiser, 1974) test for sampling adequacy and found that the sampling is sufficient, with an overall KMO value of .67, exceeding the recommended cutoff value of .60 (Kaiser, 1970, 1974). Of the 300 cases, 49 were missing values on at least one ODARA item; therefore, the final sample in the EFA was 251. The general factor showed acceptable internal consistency, with omega total and omega hierarchical values of .85 and .72, respectively.

According to the factor retention methods, all of which suggested a different number of factors to retain, we examined the 9-, 7-, and 6-factor models further. Although the 9- and 7-factor structures had good fit statistics (see Supplemental Table S1 [available in the online version of this article] for model fit statistics for models 1 through 9), many items from both models cross-loaded (i.e., loaded onto more than one factor) and contained negative residual variances (i.e., variances of the observed variables after accounting for all shared variance), suggesting that the factors were unstable and the models are not a good fit for the data. We then examined the 6-factor model, which had good fit, with RMSEA of .031 (90% CI [.022, .039]), CFI of .916, and SRMR of .076. Only five items cross-loaded in the 6-factor model and the factors appeared to be more interpretable; that is, items similar in content clustered together (see Supplemental Table S2 for factor loadings). Therefore, the 6-factor model seemed to fit these data best, and together, the factors accounted for 37.99% of the variance.

Factor 1 had poor internal consistency (ω = .58; α = .56) and consisted of six items that concern the victim; thus, we labeled this factor *Barriers to Victim Support*. Factor 2 had good internal consistency (ω = .79; α = .65) and consisted of nine items that seemed to reflect the man’s antisocial patterns and psychosocial adjustment; therefore, we named this factor *Antisocial Patterns and Psychosocial Adjustment*. Factor 3 had good internal consistency (ω = .76; α = .52) and consisted of five items specific to IPV and conditional release violations; thus, we named this factor *IPV-Specific and Technical Violations*. The two items that comprised Factor 4 reflected threats (i.e., ODARA item 5, *Threat to harm or kill at the index assault*; and SARA item 19, *Use of weapons and/or credible threats of death [index only]*); thus, we labeled this factor *Threats*, which had acceptable internal consistency (ω = .66; α = .70). The seven items that comprised Factor 5 appeared to reflect severe IPV items, denial, and negative attitudes regarding IPV; thus, we labeled this factor *Severe IPV and Negative Attitudes*, which had good internal consistency (ω = .70; α = .70). Factor 6 consisted of four items that reflect the victim’s fear of the perpetrator; thus, we named this factor *Victim Concern*, which had poor internal consistency (ω = .50; α = .53). The bivariate correlations among the six retained factors are displayed in Supplemental Table S3.

### Prediction of Recidivism Outcomes

Table 2 displays the AUC values of the six EFA factors for predicting IPV, any violent, and any recidivism. Of the 251 men with complete scores on all risk assessment measures used in the present study, 24 men were further excluded from analyses on the prediction of recidivism outcomes because eight were missing follow-up data and 16 were not released from custody or were deceased at the time of follow-up, resulting in 227 men with complete risk assessment and follow-up data. For analyses predicting IPV recidivism only, seven men were additionally excluded because information on whether they violently reoffended against a past or current partner was unavailable. Eighty (36.4%) of the remaining 220 men had a post-index IPV offense. Results indicated that only Factors 2 (*Antisocial Patterns and Psychosocial Adjustment*) and 3 (*IPV-Specific and Technical Violations*) significantly predicted IPV recidivism, with medium effect sizes. The remaining factors did not significantly predict IPV recidivism.

**Table 2:** Predictive Validities of Six Factors Retained from the Exploratory Factor Analysis for Recidivism Outcomes

Factor	AUC [95% CI]		
	IPV recidivism (*n* = 220; 80, 140)	Any violent recidivism (*n* = 227; 112, 115)	Any recidivism (*n* = 227; 148, 79)
1. Barriers to Victim Support	.53 [.45, .61]	.53 [.45, .60]	.48 [.40, .56]
2. Antisocial Patterns and Psychosocial Adjustment	.**65 [.58, .73]**	.**69 [.63, .76]**	.**71 [.64, .78]**
3. IPV-Specific and Technical Violations	.**61 [.53, .68]**	.**61 [.54, .69]**	.**67 [.60, .74]**
4. Threats	.51 [.43, .58]	.54 [.47, .62]	.53 [.45, .61]
5. Severe IPV and Negative Attitudes	.54 [.46, .62]	.54 [.46, .61]	.51 [.42, .59]
6. Victim Concern	.50 [.42, .58]	.50 [.42, .57]	.52 [.44, .60]

*Note.* Bolded values are statistically significant based on confidence interval not including .50. Sample sizes (*n*) vary because recidivism information was not available for all cases. Number of recidivists and non-recidivists in parentheses, respectively. IPV = intimate partner violence; CI = confidence interval.

For any violent recidivism, of the 227 men with complete risk assessment and follow-up data, 112 (49.3%) had a post-index violent offense. Results indicated that only Factors 2 (*Antisocial Patterns and Psychosocial Adjustment*) and 3 (*IPV-Specific and Technical Violations*) significantly predicted any violent recidivism, with medium effect sizes. The remaining factors did not significantly predict any violent recidivism.

For any recidivism, of the 227 men with complete risk assessment and follow-up data, 148 (65.2%) had at least one post-index offense. Results indicated that only Factors 2 (*Antisocial Patterns and Psychosocial Adjustment*) and 3 (*IPV-Specific and Technical Violations*) significantly predicted any reoffending, with large and medium effect sizes, respectively. The remaining factors did not significantly predict any recidivism.

### Pairwise Comparisons Among EFA Factors

We also examined whether differences between paired ROC curves (see Supplemental Figures S1–S6) among EFA factors were statistically significant for all recidivism outcomes (see Table 3). For IPV recidivism, only one significant difference emerged between Factors 2 and 6 (*D* = 2.77, *p* < .006), indicating that *Antisocial Patterns and Psychosocial Adjustment* was significantly better at predicting IPV recidivism than *Victim Concern*. For any violent recidivism, *Antisocial Patterns and Psychosocial Adjustment* significantly outperformed *Barriers to Victim Support* (*D* = 3.23, *p* < .006), *Threats* (*D* = 2.77, *p* < .006), and *Victim Concern* (*D* = 3.90, *p* < .006). For any recidivism, *Antisocial Patterns and Psychosocial Adjustment* significantly outperformed *Barriers to Victim Support* (*D* = 3.09, *p* < .006), *Threats* (*D* = 2.86, *p* < .006), *Severe IPV and Negative Attitudes* (*D* = 3.23, *p* < .006), and *Victim Concern* (*D* = 3.48, *p* < .006).

**Table 3:** Pairwise Comparisons of the Six Factors Retained from the Exploratory Factor Analysis in the Prediction of Recidivism Outcomes

Pairwise comparison	Recidivism *D*		
	IPV (*n* = 220; 80, 140)	Any violent (*n* = 227; 112, 115)	General (*n* = 227; 148, 79)
Factor 2 vs. Factor 1	1.78	3.23*	3.09*
Factor 2 vs. Factor 3	0.87	1.67	0.93
Factor 2 vs. Factor 4	2.52^†^	2.77*	2.86*
Factor 2 vs. Factor 5	1.95	3.00*	3.45*
Factor 2 vs. Factor 6	2.77*	3.90*	3.48*
Factor 3 vs. Factor 1	0.94	1.57	2.31^†^
Factor 3 vs. Factor 4	1.72	1.28	2.15^†^
Factor 3 vs. Factor 5	1.02	1.25	2.57^†^
Factor 3 vs. Factor 6	1.99^†^	2.27^†^	2.54^†^

*Note. D* values represent the statistical difference between two ROC curves, using the Bootstrap method in R. Sample sizes (*n*) vary because recidivism information was not available for all cases. Number of recidivists and non-recidivists in parentheses, respectively. Factor 1 = Barriers to Victim Support; Factor 2 = Antisocial Patterns and Psychosocial Adjustment; Factor 3 = IPV-Specific and Technical Violations; Factor 4 = Threats; Factor 5 = Severe IPV and Negative Attitudes; Factor 6 = Victim Concern.

* *p* < .006, ^†^*p* < .05.

### Incremental Validity of EFA Factors

Next, we examined whether any of the EFA factors incrementally predicted IPV and any violent recidivism over time. Among IPV recidivists (*n* = 80; 36.4%), the mean time until the first post-index IPV arrest or charge was 565 days (*SD* = 487; *Mdn* = 471). Only Factor 2 (*Antisocial Patterns and Psychosocial Adjustment*) significantly and incrementally predicted IPV recidivism over time (see Table 4), such that each point increase on Factor 2 total corresponded to an increase in the risk of IPV recidivism by 8.9%.

**Table 4:** Cox Regression Survival Analysis: Six Factors Retained From the Exploratory Factor Analysis in the Prediction of Intimate Partner Violence and Any Violent Recidivism

Predictors	*B*	*SE*	*Wald*	*p*	*e^b^*	95% CI	
						LL	UL
	Intimate partner violence recidivism (*n* = 220)						
1. Barriers to Victim Support	.016	.043	0.144	.705	1.016	0.935	1.105
2. Antisocial Patterns and Psychosocial Adjustment	.085	.035	6.010	.**014**	1.089	1.017	1.166
3. IPV-Specific and Technical Violations	.071	.054	1.684	.194	1.073	0.965	1.194
4. Threats	.049	.078	0.402	.526	1.051	0.902	1.224
5. Severe IPV and Negative Attitudes	–.033	.040	0.699	.403	0.967	0.895	1.046
6. Victim Concern	–.050	.060	0.677	.411	0.952	0.845	1.071
	Any violent recidivism (*n* = 227)						
1. Barriers to Victim Support	.002	.036	0.002	.962	1.002	0.933	1.075
2. Antisocial Patterns and Psychosocial Adjustment	.125	.031	16.154	**<.001**	1.133	1.066	1.205
3. IPV-Specific and Technical Violations	.074	.047	2.482	.115	1.077	0.982	1.181
4. Threats	.038	.066	0.326	.568	1.038	0.913	1.181
5. Severe IPV and Negative Attitudes	–.039	.034	1.335	.248	0.962	0.900	1.028
6. Victim Concern	–.059	.052	1.271	.260	0.943	0.851	1.045

*Note.* Significant *p*-values in bold. Sample sizes (*n*) vary because recidivism information was not available for all cases. For intimate partner violence recidivism, *n* = 80 recidivists and *n* = 140 non-recidivists. For any violent recidivism, *n* = 112 recidivists and *n* = 115 non-recidivists.

For any violent recidivism, 112 (49.3%) recidivated and the mean time until their first post-index arrest or charge for any violent offense was 505 days (*SD* = 480; *Mdn* = 384). Like IPV recidivism, only Factor 2 (*Antisocial Patterns and Psychosocial Adjustment*) significantly and incrementally predicted any violent recidivism over time (Table 4), such that each additional point on Factor 2 total was associated with a 13.3% increased risk of any violent recidivism.

## Discussion

Our factor analysis of the ODARA, SARA, and B-SAFER revealed that IPV risk is multidimensional, with at least six latent constructs, which is consistent with past research (Hilton, Harris, & Rice, 2010; Pham & Jung, 2022). As expected and consistent with past research (e.g., Hilton, Harris, & Rice, 2010; Pham & Jung, 2022), Factors 2 (*Antisocial Patterns and Psychosocial Adjustment*) and 3 (*IPV-Specific and Technical Violations*) significantly predicted all recidivism outcomes in the current study. Factor 2 was also statistically better at predicting any violent and any recidivism than Factors 1 (*Barriers to Victim Support*), 4 (*Threats*), 5 (*Severe IPV and Negative Attitudes*), and 6 (*Victim Concern*). Factor 2 contained more items than the other factors, arguably potentially giving it an advantage over the other factors when predicting recidivism outcomes. This seems unlikely, however, given that Factor 3—which only contained five items—also significantly predicted recidivism outcomes. The more likely explanation is that Factor 2 contained seven of the eight items from the Central Eight risk factors (i.e., criminal history, procriminal peers/associates, antisocial personality pattern, school/work problems, substance use), which consistently and significantly predict general criminal and violent recidivism and represent the most predictive risk factors for criminal activities (e.g., Bonta & Andrews, 2024; Goldstein et al., 2016; Olver et al., 2014). Indeed, within the IPV literature, scholars have begun to examine men’s use of IPV through the lens of the RNR principles of effective correctional service (e.g., Bonta & Andrews, 2024; Richards et al., 2021) and found that RNR adherence is positively associated with treatment effectiveness (Travers et al., 2021).

Surprisingly, Factor 6 (*Victim Concern*) did not significantly predict any recidivism outcomes in the current study. By contrast, prior research has demonstrated women’s ability to assess their own risk of IPV revictimization (e.g., Bell & Naugle, 2008; Connor-Smith et al., 2011) and van der Put et al.’s (2019) recent meta-analysis found that victims predicted new offenses at levels comparable to some IPV risk assessment measures (AUC = .64, *k* = 7). Furthermore, none of the other IPV-specific factors (i.e., Factors 1, 4–6) significantly predicted IPV recidivism, which further begs the question of whether specialized IPV risk assessment measures are necessary. Indeed, Factor 2 significantly and independently predicted any violent and IPV recidivism over time above and beyond all other factors, suggesting that the latter factors are redundant for violence prediction, further confirming the importance of the Central Eight factors in predicting violence. To date, the exploration of RNR in relation to IPV is relatively new and substantial questions remain (Radatz et al., 2021; Radatz & Hilton, 2025), highlighting the importance of investigating specialized IPV risk assessment measures.

We have previously commented on the difficulty of coding mental health and victim-vulnerability variables garnered during police investigations, which may result in failure to identify relationships between these variables and recidivism. Better information could facilitate more accurate coding of IPV- and victim-specific risk variables and permit a fairer test of their predictive validity. It is also possible that some risk factors are more relevant for specific perpetrator subtypes than for others. There is substantial evidence that men who engage in IPV are a heterogenous group who differ on general criminality and violence, severity of violence, antisocial and psychopathic traits, personality traits, weapon use, victim injury, and likelihood of recidivism (Holtzworth-Munroe & Meehan, 2004; Holtzworth-Munroe & Stuart, 1994; Peters et al., 2023). Indeed, violence severity may only be predictive of IPV recidivism for men who are generally violent/antisocial but not for those who exclusively target their family members (Goldstein et al., 2016), suggesting that the relationship between risk factors and IPV recidivism may depend on perpetrator subtype. Similarly, it is reasonable to hypothesize that Hilton, Harris, and Rice’s (2010) third factor comprising IPV-related technical violations might be more relevant for IPV offenders with personality problems related to anxious attachment (e.g., borderline/dysphoric abusers: Holtzworth-Monroe & Stuart, 1994) than for abusers with fewer emotional and behavioral self-management issues. Future research should assess the moderating effects of perpetrator subtype on the relationship between risk factors and IPV recidivism.

### Limitations

Our sample size was smaller than previously recommended for factor analysis and tests of incremental validity (e.g., Comrey & Lee, 1992; Tabachnick & Fidell, 2019), which reduced statistical power and stability of our results. However, the KMO test for sampling adequacy revealed an overall value of .67, suggesting that the sampling is sufficient (Kaiser, 1970, 1974). It is also generally suggested that factors with a large number of high factor loadings are reliable regardless of sample size (Guadagnoli & Velicer, 1988). Our factors contained several items with high factor loadings (e.g., .50 and above; see Supplemental Table S2), suggesting that the factors may be reliable despite the small sample size. Interrater reliability was lower for items that assessed psychological constructs and victim concern (Hilton et al., 2021), which could have rendered the underlying factors invalid or unstable and reduced the predictive validity of the factors reported. Furthermore, this study was conducted with high-risk men in one specific law enforcement organization in Canada (see Ennis et al., 2015; Hilton et al., 2021); therefore, generalizability to other populations, jurisdictions, or agencies cannot be assumed. We also had limited information to examine the data from an intersectional perspective. A growing area of focus is intersectionality (e.g., Batastini et al., 2022), which examines how a person’s overlapping identities, such as gender, sexuality, race, and socioeconomic status intersect to shape their lived experiences, and potentially their responsiveness to intervention. Future research should attempt to replicate these findings through the lens of intersectionality, using a larger sample from other organizations with additional sources of data (i.e., interviews).

Given the high-risk nature of our sample, many men were likely subject to relatively intensive management efforts. If such efforts reduced their likelihood of recidivism, this could have attenuated the predictive validities found in the present study. Moreover, individuals who are arrested, charged, or convicted in relation to a general or other non-IPV crime may no longer be at risk for IPV while in custody for these crimes (i.e., competing risks of recidivism; Noordzij et al., 2013). However, we could not account for competing risks of recidivism because we did not collect the required information. Future research should attempt to replicate these findings while accounting for competing risks of recidivism, as well as investigate potential moderating effects of risk management interventions on the relationship between risk and recidivism.

## Conclusion

Our findings suggest that there are multiple dimensions that comprise IPV risk, directing professionals toward further potential needs of men who engage in IPV. If future research replicates these findings and demonstrates a similar number and nature of these domains, then IPV interventions could eventually be tailored to focus on the risk domains most strongly associated with IPV recidivism, in line with RNR principles. The extent to which correctional services address characteristics that are most strongly related to offending behavior significantly affects their treatment success. Evidently, more work is needed to improve our current understanding of IPV risk, particularly the need for theoretically informed risk assessments.

## Supplemental Material

sj-docx-1-cjb-10.1177_00938548251357789 – Supplemental material for The Factor Structure of Intimate Partner Violence Risk Supplemental material, sj-docx-1-cjb-10.1177_00938548251357789 for The Factor Structure of Intimate Partner Violence Risk by Anna T. Pham, Kevin L. Nunes, N. Zoe Hilton, Liam Ennis and Sandy Jung in Criminal Justice and Behavior
