# Supplementary material for: The Factor Structure of Intimate Partner Violence Risk
Source: Crim Justice Behav. 2025 Sep 1;53(1):101–18. doi: 10.1177/00938548251357789 (PMC12701759; doi:10.1177/00938548251357789)
Supplement: sj-docx-1-cjb-10.1177_00938548251357789 – Supplemental material for The Factor Structure of Intimate Partner Violence Risk [file sj-docx-1-cjb-10.1177_00938548251357789.docx]

**Supplemental Materials**

**Table S1**

*Model Fit Statistics of Models 1 Through 9 for the Exploratory Factor Analysis*

| Model | RMSEA (90% CI) | CFI | SRMR |
| --- | --- | --- | --- |
| 1-factor | .066 (.061, .071) | .496 | .143 |
| 2-factor | .055 (.049, .060) | .669 | .119 |
| 3-factor | .048 (.042, .054) | .764 | .105 |
| 4-factor | .042 (.036, .049) | .824 | .096 |
| 5-factor | .036 (.028, .043) | .880 | .084 |
| 6-factor | .031 (.022, .039) | .916 | .076 |
| 7-factor | .025 (.013, .035) | .949 | .068 |
| 8-factor | .018 (.000, .030) | .975 | .059 |
| 9-factor | .014 (.000, .027) | .987 | .054 |

*Note*. RMSEA (Root Mean Square Error of Approximation) should be less than .06, CFI (Comparative Fit Index) should be .95 or greater, and SRMR (Standardized Root Mean Square Residual) should be less than .08 to indicate good fit.

**Table S2**

*Rotated Factor Loadings and Standardized Rotated Loadings for the 6-Factor Model of All Items Included in the Exploratory Factor Analysis*

|  | Rotated Factor Loading  (Standardized Rotated Factor Loading) | | | | | |
| --- | --- | --- | --- | --- | --- | --- |
| Ontario Domestic Assault Risk Assessment | Factor 1 | Factor 2 | Factor 3 | Factor 4 | Factor 5 | Factor 6 |
| 1. Prior domestic incident | **.33**  (2.67) | .12  (0.54) | **.54**  (3.37) | .15  (0.92) | .27  (1.66) | -.06  (-0.08) |
| 1. Prior non-domestic incident | .01  (0.11) | **.61**  **(5.23)** | .21  (0.90) | .01  (0.10) | -.16  (-1.47) | -.02  (-0.18) |
| 1. Prior custodial sentence of 30 days or more | .08  (0.93) | **.69**  **(4.81)** | **.47**  (1.80) | -.07  (-0.93) | -.11  (-1.06) | .01  (0.16) |
| 1. Failure on prior conditional release | -.11  (-0.82) | **.53**  (3.13) | **.72**  (3.44) | .04  (0.62) | .04  (0.62) | -.05  (-0.62) |
| 1. Threat to harm or kill at the index assault | -.08  (-1.14) | .00  (-0.01) | -.02  (-0.34) | **.80**  **(9.47)** | .13  (0.99) | .07  (0.94) |
| 1. Confinement of the victim at the index assault | -0.27  (-2.65) | .03  (0.34) | .07  (0.67) | .03  (0.31) | .08  (0.83) | .27  (2.70) |
| 1. Victim concern about future assaults | -.038  (-0.71) | -.004  (-0.07) | -.07  (-0.77) | .24  (2.10) | -.15  (-1.15) | **.88**  **(9.34)** |
| 1. More than one child | **.70**  **(8.43)** | -.14  (-1.28) | .021 (0.29) | -.01  (-0.18) | .01  (0.09) | -.02  (-0.24) |
| 1. Victim’s biological child from a previous partner | **.52**  **(6.24)** | -.04  (-0.44) | .17  (1.65) | -.05  (-0.67) | -.15  (-1.37) | .18  (1.78) |
| 1. Two or more indicators of substance abuse | .12  (1.07) | **.47**  **(4.08)** | .11  (0.52) | -.09  (-0.78) | -.02  (-0.20) | -.12  (-1.03) |
| 1. Assault on the index victim when she was pregnant | **.31**  (3.00) | .07  (0.75) | -.05  (-0.47) | .001  (0.01) | **.39**  **(3.83)** | -.23  (-1.96) |
| 1. Barriers to victim support | **.72**  **(7.32)** | -.17  (-1.28) | .03  (0.47) | -.08  (-0.78) | -.09  (-0.77) | -.03  (-0.35) |
| Spousal Assault Risk Assessment |  |  |  |  |  |  |
| 1. Past assault of family members | **.33**  (3.40) | **.50**  **(5.63)** | -.08  (-0.42) | .14  (1.55) | .02  (0.36) | -.06  (-0.74) |
| 1. Recent relationship problems | .06  (0.55) | -.14  (-1.17) | .06  (0.59) | -.04  (-0.40) | .21  (1.76) | **.34**  (3.14) |
| 1. Victim of and/or witness to family violence as a child or adolescent | .10  (1.11) | **.31**  (3.34) | -.15  (-1.01) | .004  (0.05) | .09  (0.86) | .09  (0.84) |
| 1. Recent suicidal or homicidal ideation/intent | .06  (0.65) | -.04  (-0.43) | .03  (0.32) | .23  (2.75) | -.03  (-0.37) | .21  (2.39) |
| 1. Recent psychotic and/or manic symptoms | -.15  (-1.20) | .03  (0.28) | -.02  (-0.13) | .16  (1.23) | .04  (0.31) | .26  (1.98) |
| 1. Personality disorder with anger, impulsivity, or behavioral instability | -.07  (-0.84) | **.40**  **(4.96)** | -.05  (-0.37) | -.05  (-0.67) | .13  (1.32) | .29  (3.07) |
| 1. Past sexual assault/sexual jealousy | .09  (1.00) | -.19  (-2.14) | .21  (1.84) | -.13  (-1.41) | **.41**  (3.48) | .12  (1.29) |
| 1. Past use of weapons and/or credible threats of death | .001  (0.01) | .02  (0.22) | .28  (2.53) | .08  (0.81) | **.32**  (2.84) | .15  (1.48) |
| 1. Past violation of “no contact” orders | -.02  (-0.32) | .01  (0.05) | **.76**  **(8.32)** | -.05  (-0.68) | .22  (1.29) | -.002  (-0.03) |
| 1. Extreme minimization or denial of spousal assault history | .02  (0.34) | -.16  (-2.16) | .001  (0.10) | .002  (0.02) | **.62**  **(9.00)** | -.02  (-0.24) |
| 1. Attitudes that support or condone spousal assault | .01  (0.20) | -.02  (-0.48) | -.19  (-1.16) | .002  (0.04) | **.92**  **(16.46)** | -.28  (-2.83) |
| 1. Severe and/or sexual assault | -.09  (-0.94) | .04  (0.39) | **.31**  (3.55) | .07  (0.85) | -.002  (-0.03) | .08  (0.78) |
| 1. Use of weapons and/or credible threats of death | .04  (0.89) | -.04  (-0.72) | .19  (1.59) | **.98**  **(13.07)** | -.07  (-0.78) | -.04  (-0.90) |
| 1. Violation of “no contact” order | .04  (0.45) | -.04  (-0.38) | **.45**  **(4.96)** | .04  (0.47) | .03  (0.35) | .28  (2.77) |
| Brief Spousal Assault Form for the Evaluation of Risk |  |  |  |  |  |  |
| 1. Violent threats or thoughts | -.01  (-0.11) | .05  (0.53) | .12  (0.91) | .23  (2.21) | **.50**  **(4.21)** | .15  (1.24) |
| 1. Escalation | .12  (1.14) | -.12  (-1.40) | .06  (0.64) | .16  (1.82) | .17  (1.75) | .25  (2.90) |
| 1. Violent attitudes | -.04  (-0.88) | .003  (0.06) | .07  (0.43) | -.06  (-0.93) | **.93**  **(18.06)** | .03  (0.46) |
| 1. General criminality | -.02  (-0.42) | **.70**  **(7.18)** | .15  (0.61) | -.17  (-2.26) | -.01  (-0.09) | .07  (0.81) |
| 1. Employment problems | .17  (1.71) | **.41**  **(4.59)** | .08  (0.51) | .04  (0.47) | .05  (0.55) | .06  (0.61) |
| 1. Mental health problems | -.02  (-0.19) | **.34**  (3.63) | -.23  (-1.74) | .03  (0.44) | .10  (0.78) | .22  (2.02) |
| 1. Inconsistent attitudes or behaviour | **.41**  **(4.84)** | .09  (0.86) | .16  (1.32) | .01  (0.18) | .10  (0.95) | .14  (1.48) |
| 1. Extreme fear of perpetrator | .19  (1.84) | .00  (0.003) | -.01  (-0.09) | -.06  (-0.83) | .03  (0.50) | **.85**  **(13.68)** |
| 1. Inadequate support or resources | **.46**  **(5.05)** | .05  (0.69) | -.25  (-2.09) | .14  (1.41) | .21  (1.67) | .11  (0.95) |
| 1. Unsafe living situation | .25  (2.61) | .06  (0.66) | -.17  (-1.74) | -.05  (-0.69) | .05  (0.49) | **.34**  (3.57) |
| 1. Health problems | **.50**  **(6.29)** | .01  (0.17) | -.10  (-0.91) | .13  (1.35) | .06  (0.57) | -.04  (-0.48) |

*Note.* Unstandardized factor loadings greater than .30 and standardized factor loadings greater than 3.65 (critical z-score), α = .00013 are considered significant. Bolded values indicate significant loading on the factor. Factor 1 = Barriers to Victim Support; Factor 2 = Antisocial Patterns and Psychosocial Adjustment; Factor 3 = IPV-Specific and Technical Violations; Factor 4 = Threats; Factor 5 = Severe IPV and Negative Attitudes; Factor 6 = Victim Concern.

**Table S3**

*Bivariate Correlations Between the Six Factors Retained from the Exploratory Factor Analysis*

| Factor | Factor 1 | Factor 2 | Factor 3 | Factor 4 | Factor 5 | Factor 6 |
| --- | --- | --- | --- | --- | --- | --- |
| Factor 1: Barriers to Victim Support | - | .12 | .11 | .01 | **.28***** | .11 |
| Factor 2: Antisocial Patterns and Psychosocial Adjustment | **.13*** | - | **.24***** | .01 | **.18**** | .06 |
| Factor 3: IPV-Specific and Technical Violations | .10 | **.26***** | - | .03 | **.13*** | .06 |
| Factor 4: Threats | .01 | .01 | .01 | - | **.16*** | **.14*** |
| Factor 5: Severe IPV and Negative Attitudes | **.29***** | **.17**** | **.13*** | **.16*** | - | **.41***** |
| Factor 6: Victim Concern | .11 | .07 | .06 | .12 | **.38***** | - |

*Note*. *N* = 251. Pearson’s *r* (parametric) correlations above the diagonal and Spearman’s rho (non-parametric) correlations below the diagonal and are based on factor scores generated from the exploratory factor analysis in Mplus.

* *p* < .05, ** *p* < .01, *** *p* < .001.

**Figure S1**

*ROC Plots Comparing Factor 2 to All Other Factors in the Prediction of Intimate Partner Violence Recidivism*

***Notes***. Receiver operating characteristic (ROC) plots are based on factor scores. Factor 1 = Barriers to Victim Support; Factor 2 = Antisocial Patterns and Psychosocial Adjustment; Factor 3 = IPV-Specific and Technical Violations; Factor 4 = Threats; Factor 5 = Severe IPV and Negative Attitudes; Factor 6 = Victim Concern.

**Figure S2**

*ROC Plots Comparing Factor 3 to All Other Factors in the Prediction of Intimate Partner Violence Recidivism*

***Notes*.** Receiver operating characteristic (ROC) plots are based on factor scores. Factor 1 = Barriers to Victim Support; Factor 2 = Antisocial Patterns and Psychosocial Adjustment; Factor 3 = IPV-Specific and Technical Violations; Factor 4 = Threats; Factor 5 = Severe IPV and Negative Attitudes; Factor 6 = Victim Concern.

**Figure S3**

*ROC Plots Comparing Factor 2 to All Other Factors in the Prediction of Any Violent Recidivism*

***Notes***. Receiver operating characteristic (ROC) plots are based on factor scores. Factor 1 = Barriers to Victim Support; Factor 2 = Antisocial Patterns and Psychosocial Adjustment; Factor 3 = IPV-Specific and Technical Violations; Factor 4 = Threats; Factor 5 = Severe IPV and Negative Attitudes; Factor 6 = Victim Concern.

**Figure S4**

*ROC Plots Comparing Factor 3 to All Other Factors in the Prediction of Any Violent Recidivism*

***Notes*.** Receiver operating characteristic (ROC) plots are based on factor scores. Factor 1 = Barriers to Victim Support; Factor 2 = Antisocial Patterns and Psychosocial Adjustment; Factor 3 = IPV-Specific and Technical Violations; Factor 4 = Threats; Factor 5 = Severe IPV and Negative Attitudes; Factor 6 = Victim Concern.

**Figure S5**

*ROC Plots Comparing Factor 2 to All Other Factors in the Prediction of General Recidivism*

***Notes***. Receiver operating characteristic (ROC) plots are based on factor scores. Factor 1 = Barriers to Victim Support; Factor 2 = Antisocial Patterns and Psychosocial Adjustment; Factor 3 = IPV-Specific and Technical Violations; Factor 4 = Threats; Factor 5 = Severe IPV and Negative Attitudes; Factor 6 = Victim Concern.

**Figure S6**

*ROC Plots Comparing Factor 3 to All Other Factors in the Prediction of General Recidivism*

***Notes*.** Factor 1 = Barriers to Victim Support; Factor 2 = Antisocial Patterns and Psychosocial Adjustment; Factor 3 = IPV-Specific and Technical Violations; Factor 4 = Threats; Factor 5 = Severe IPV and Negative Attitudes; Factor 6 = Victim Concern.
